# Supplementary material for: Staphylococcus aureus functional amyloids catalyze degradation of β-lactam antibiotics
Source: Nat Commun. 2023 Dec 11;14:8198. doi: 10.1038/s41467-023-43624-1 (PMC10713593; doi:10.1038/s41467-023-43624-1)
Supplement: Supplementary file 1 — Supplementary Information [file 41467_2023_43624_MOESM1_ESM.pdf]

ASSOCIATED CONTENT

Staphylococcus aureus functional amyloids catalyze degradation of β-lactam antibiotics

*Elad Arad, Kasper B. Pedersen, Orit Malka, Sisira Mambram Kunnath, Nimrod Golan, Polina Aibinder, Birgit Schiøtt, Hanna Rapaport, Meytal Landau and Raz Jelinek**

Table of Contents

The following files are available free of charge.

Figure S1. FTIR spectroscopy of PSMα1-4.

Figure S2. Amyloid-β 1-42 fibrils reaction with nitrocefin.

Figure S3. Cross-β bacterial amyloids of *Pseudomonas Aeruginosa* reaction with nitrocefin.

Figure S4. Critical dissolution concentration of PSMα1-4.

Figure S5. Recyclability of PSMα assemblies.

Table S1. Catalytic parameters of PSMα1-4 amyloids.

Figure S6. Amytracker-680 fluorescent labelling of PSMα2-3 derivatives.

Figure S7. Initial nitrocefin-degradation reaction rate in presence of PSMα3 and derivatives.

Figure S8. Catalytic activity of PSMα2 and derivatives.

Figure S9. Circular Dichroism and FTIR spectroscopy of PSMα3 point mutated derivatives.

Figure S10. The effect of the pH on the catalytic activity of PSMα3.

Figure S11. Starting conformation of the nitrocefin in MD simulations.

Figure S12. Implied timescale of the stationary process for a range of Bayesian Markov State Models with increasing lag times.

Table S2. LC Gradient program for Amoxicillin.

Table S3. LC Gradient program for Penicillin-G

Figure S13. Amoxicillin and degraded Amoxicillin LC-MS chromatogram and mass spectra

Figure S14. Penicillin and degraded Amoxicillin LC-MS chromatogram and mass spectra

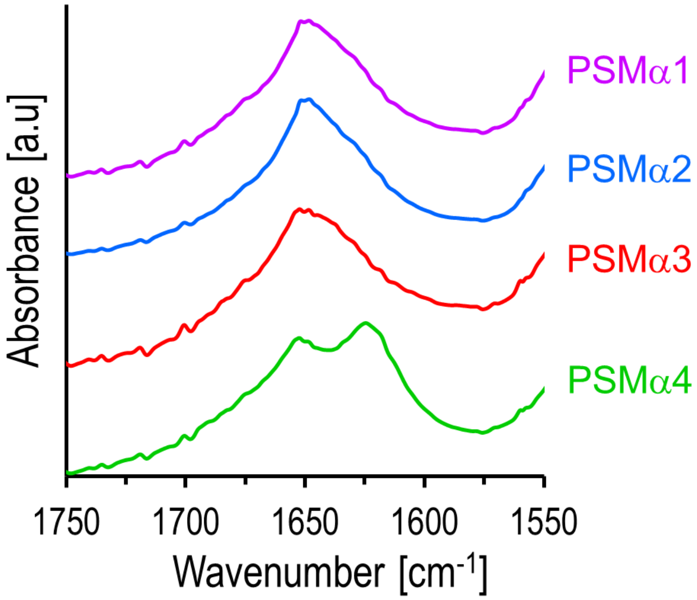

Figure S1. Fourier transform infrared spectroscopy of PSMα1-4.

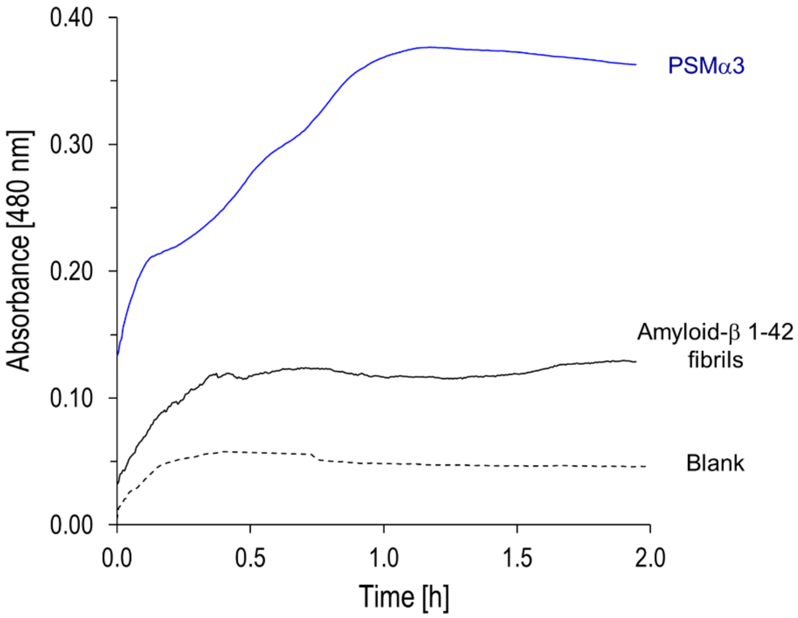

Figure S2. Amyloid-β 1-42 fibrils reaction with nitrocefin. Amyloid-β (sequence 1-42 a.a, Aβ42) was incubated for fibrillation over 24 hours at 37ºC at HEPES buffer (50 mM)[1]. 95 µL of the pre-assembled Aβ42 (100 µM) were mixed with 5 µL of concentrated nitrocefin (3.4 mM in stock, 1:1 v/v DIW/acetonitrile) to final nitrocefin concentration of 170 µM, and the absorbance at 480 nm was recorded as described earlier for two hours. In comparison, pre-assembled PSMα3 (90 µM) have resulted much higher activity at similar conditions. It should be emphasized that PSMα3 has much shorter sequence than Aβ42 (21 a.a vs. 42 a.a). This is representative result of three independent repeats of the experiments.

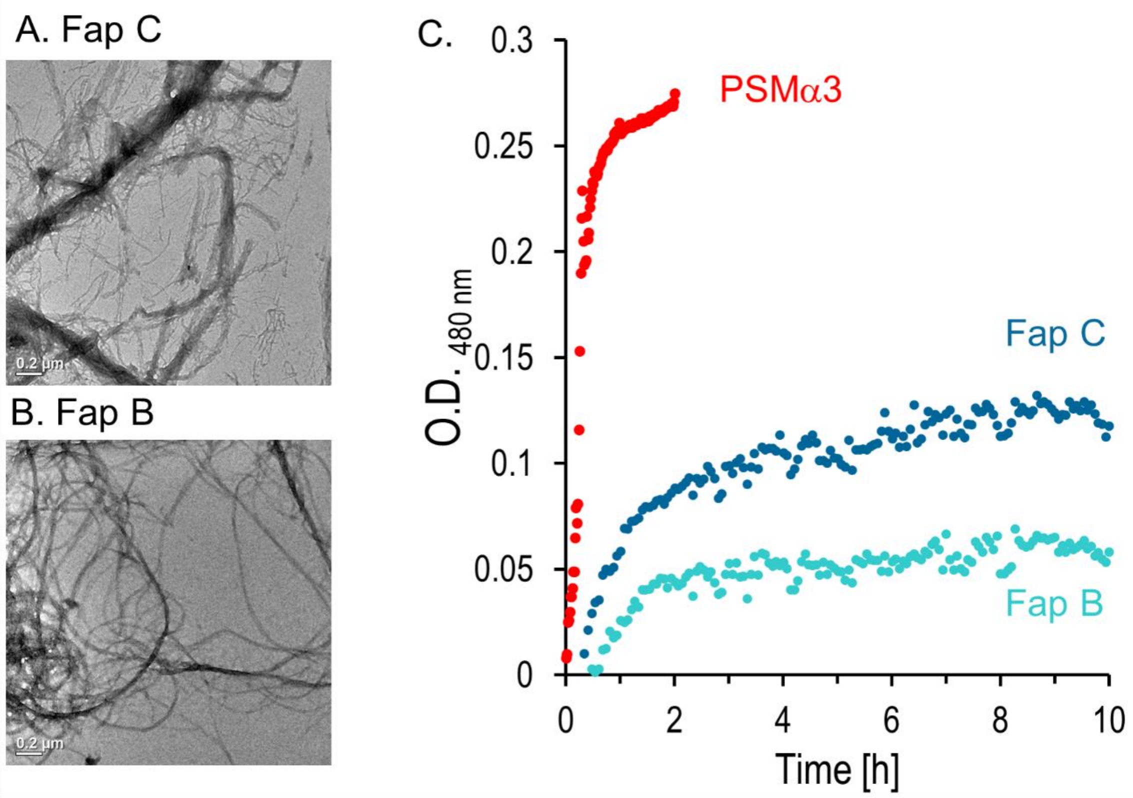

Figure S3. Cross-β bacterial amyloids of *Pseudomonas Aeruginosa*. Fap B and Fap C were incubated for seven days at 37ºC for fibrillation. Then, upon appearance of typical turbidity the samples were diluted and nitrocefin was added to the samples, creating solutions of 20 μM Fap and nitrocefin in concentration of 100 μM. The absorbance of the nitrocefin was recorded in microplate reader at 480 nm for ten hours, resulting low absorbance relatively to the PSMα at close mass concentration (w/v, 300 μM). This comparison was necessary due to the major differences in sequence length of the Fap proteins and the short PSMα3 peptide. The results are shown as absorbance rather than product concentration due to the slight turbidity of the Fap samples.

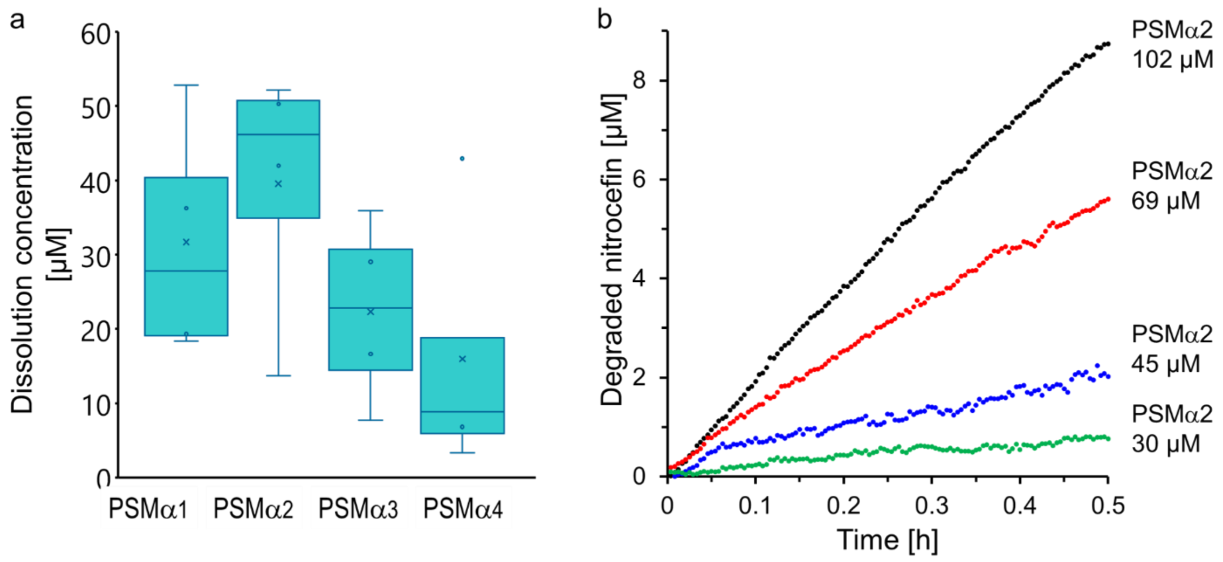

Figure S4. Critical dissolution concentration of PSMα1-4. A. The critical dissolution concentration was measured using ANS fluorescent labelling. The PSMα peptides were assembled as described above, incubated for two hours in DIW and buffered with HEPES at PSMα concentration of 440 μM. After the incubation the samples were diluted serially with the same buffer (HEPES 50 mM, pH 7.4) by 2/3 each time. PSMα solution (55 μL) was mixed with ANS aqueous solution (5 μL, 0.02 mg/mL) and placed in 384 well black plate. The samples were incubated for 30 minutes and then fluorescent measurement of ANS was applied (Excitation at 380 nm, emission 500 nm) at Biotek Synergy H1 plate reader (Biotek, Winooski, VT, USA). The fluorescence was analyzed as a function of the peptide concentration (in logarithmic scale). The critical concentration was determined as the point in which the slope of the fluorescence increases dramatically (the intercept of two linear trends of fluorescence as function of the peptide concentration)[2]. Data presented in box-and-whisker plot, with mean-line and quartile calculation using inclusive median, N=4. **B**. Nitrocefin degradation in presence of PSMα2 (below and above the critical dissolution concentration, nitrocefin concentration 60 μM).

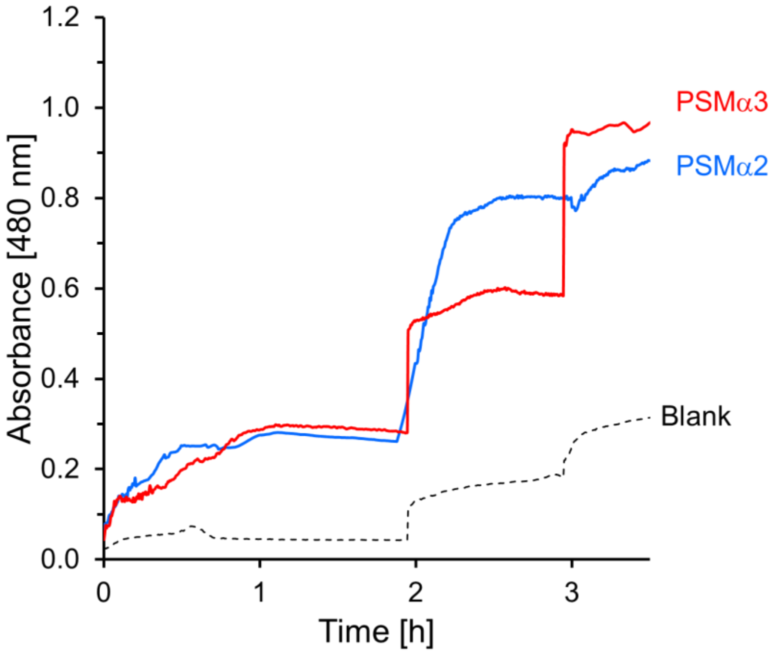

Figure S5. Recyclability test of PSMα assemblies. PSMα2 assemblies were prepared as described above and diluted to concentration of 90 µM. 5 µL of concentrated nitrocefin (3.4 mM in stock, 1:1 v/v DIW/acetonitrile) were added to 95 µL of the PSMα solution, to final nitrocefin concentration of 170 µM, and the absorbance at 480 nm was recorded as described earlier. After two hours, 5 µL of fresh nitrocefin was added and the absorbance was recorded similarly for one hour, following another addition of 5 µL of fresh nitrocefin and kinetic measurement for 30 minutes. Blank sample contained HEPES buffer.

Table S1. Catalytic parameters of PSMα1-4 amyloid assemblies

	R^2	K_M [mM]	V_{max} [μM/h]	K_{cat} $*10^4$ [1/s]	K_{cat}/K_M $[M^{-1}s^{-1}]$
PSMα1	0.95	0.2±0.2	60±30	0.9±0.5	0.4±0.2
PSMα2	0.99	0.23±0.08	240±50	3.9±0.8	1.7±0.6
PSMα3	0.96	0.08±0.03	100±20	1.6±0.3	2.1±0.7
PSMα4	0.98	0.2±0.2	80±50	1.4±0.8	0.6±0.2

Values derive from the fitting of the initial rates to MM model and are presented as *calculated-value ± confidence interval*. K_M is the Michalis constant; V_{max} is the maximal rate of reaction; k_{cat} is the turnover number, and the catalytic efficiency is $\varepsilon=K_{cat}/K_M$.

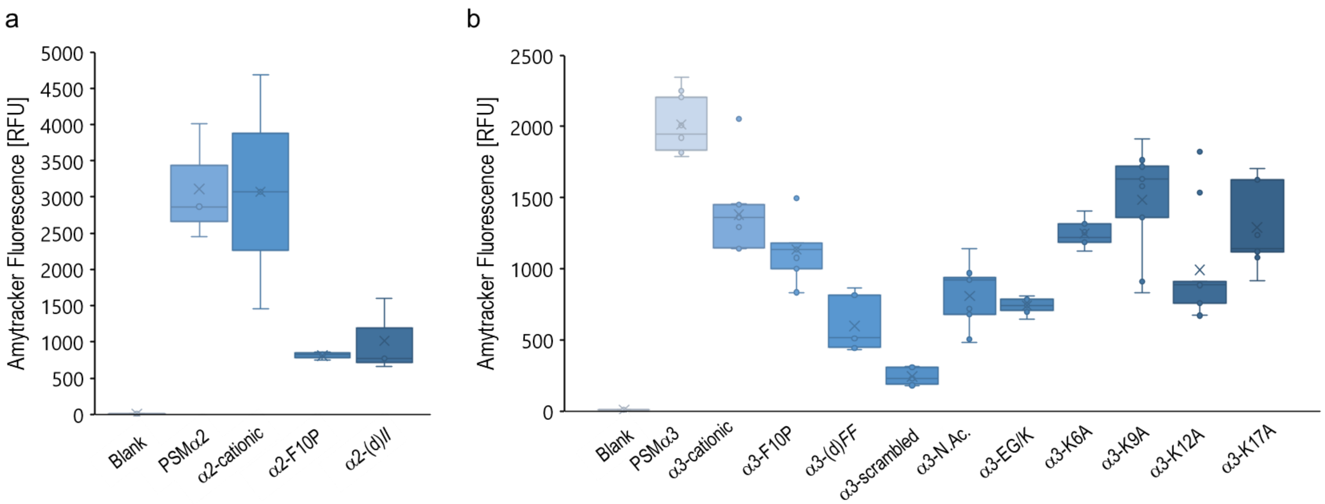

Figure S6. Amytracker-680 fluorescent labelling of PSMα2 (A) and PSMα3 (B) derivatives.

Amyloid staining with *Amytracker 680* (excitation 552 nm, emission 654 nm, PSMα concentrations were 400 μM). Data presented in box-and-whisker plot, with mean line and quartile calculation using inclusive median, $N_{PSM\alpha2\ derivatives}$=3, $N_{PSM\alpha3\ derivatives}$=9.

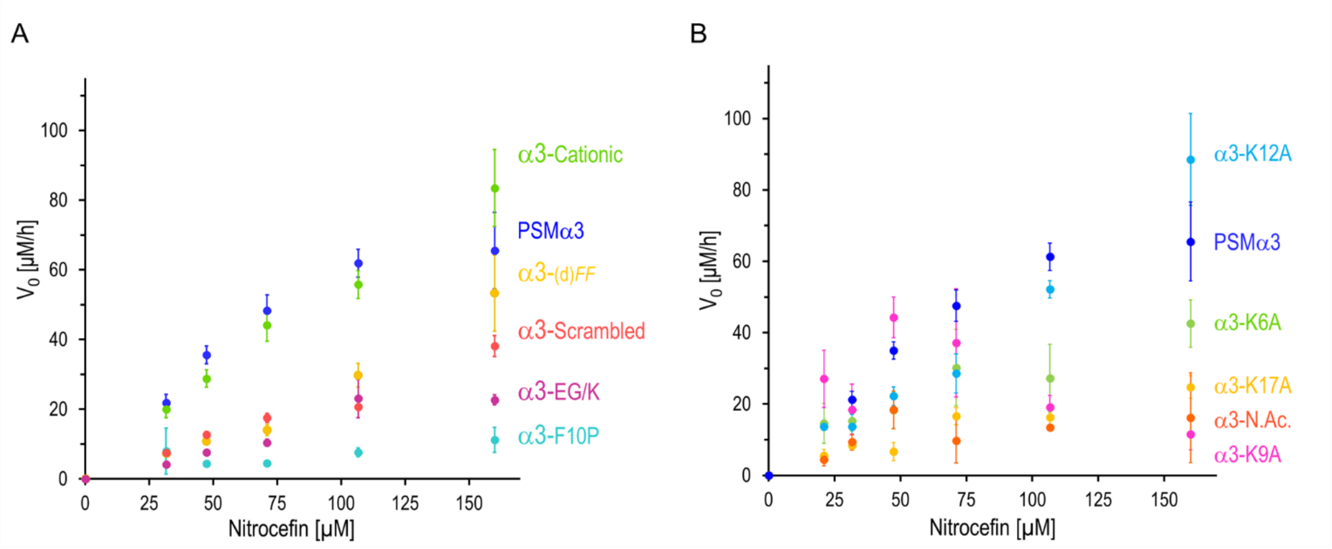

Figure S7. Initial nitrocefin-degradation reaction rate in presence of PSMα3 and derivatives. Initial nitrocefin-degradation reaction rate, V_0, as a function of initial substrate concentration in the presence of PSMα3 peptide assemblies (or derivatives, 170 μM). **A.** Structural derivatives. **B.** Point mutation derivatives. The results are presented as average±SEM, N=3.

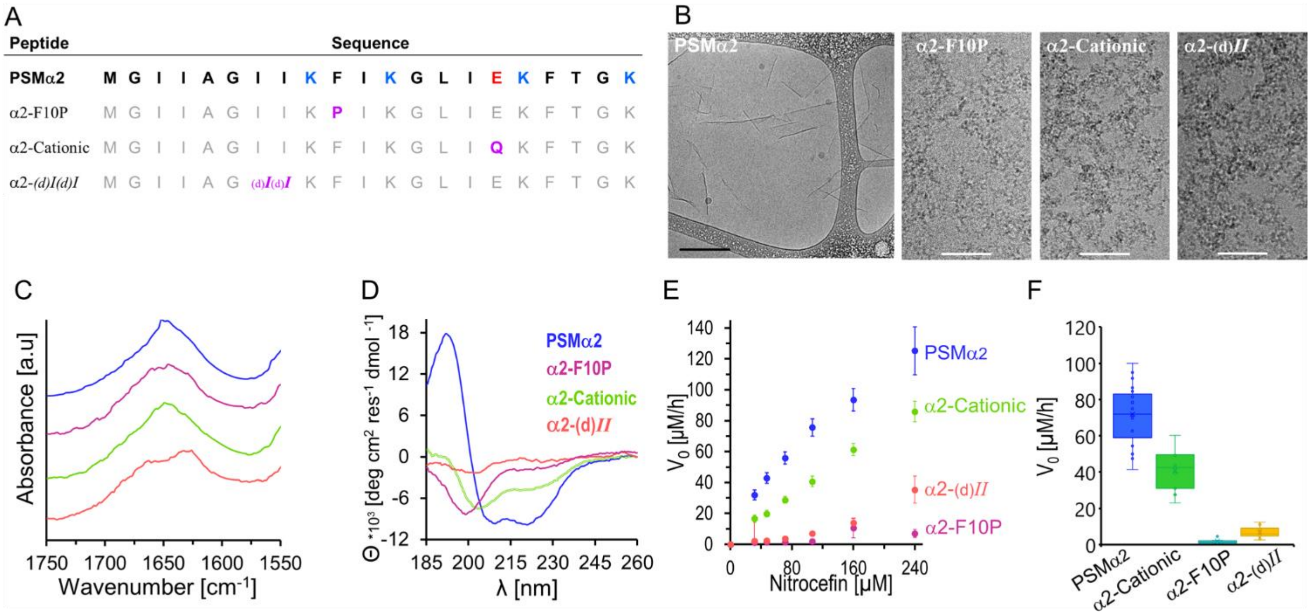

Figure S8. Catalytic activity of PSMα2 and derivatives. A. PSMα2 derivative sequences. The modified residues in the derivatives are in purple. At α2-cationic also the C-terminus is amidated. For α2-(d)*II* the two Ile residues are in (D) chirality rather than (L) like the other residues. **B.** Cryo-TEM images of PSMα2 derivatives (300μM). Only PSMα2 generated sheet morphology, while the others formed only amorphous structures. Black bar correspond to 500 nm and white bars correspond to 100 nm. **C.** Fourier transform infrared spectroscopy (FTIR) of amide vibration of PSMα2 derivatives. **D.** CD spectroscopy analysis of PSMα2 derivatives. **E.** Initial nitrocefin-degradation reaction rate, V_0, as a function of initial substrate concentration in the presence of PSMα2 peptide assemblies (or derivatives, 170 μM). **F.** Initial degradation rate, V_0, at initial nitrocefin concentration of 107 μM, in presence of PSMα2 and derivatives. Data presented in box-and-whisker plot, with mean line and quartile calculation using inclusive median, $N_{PSM\alpha2}$=19, $N_{\alpha2\text{-cationic}}$=11, $N_{\alpha2\text{-F10P}}$=7, $N_{\alpha2\text{-(d)II}}$=11.The same color-coding was used for the PSMα2 derivatives.

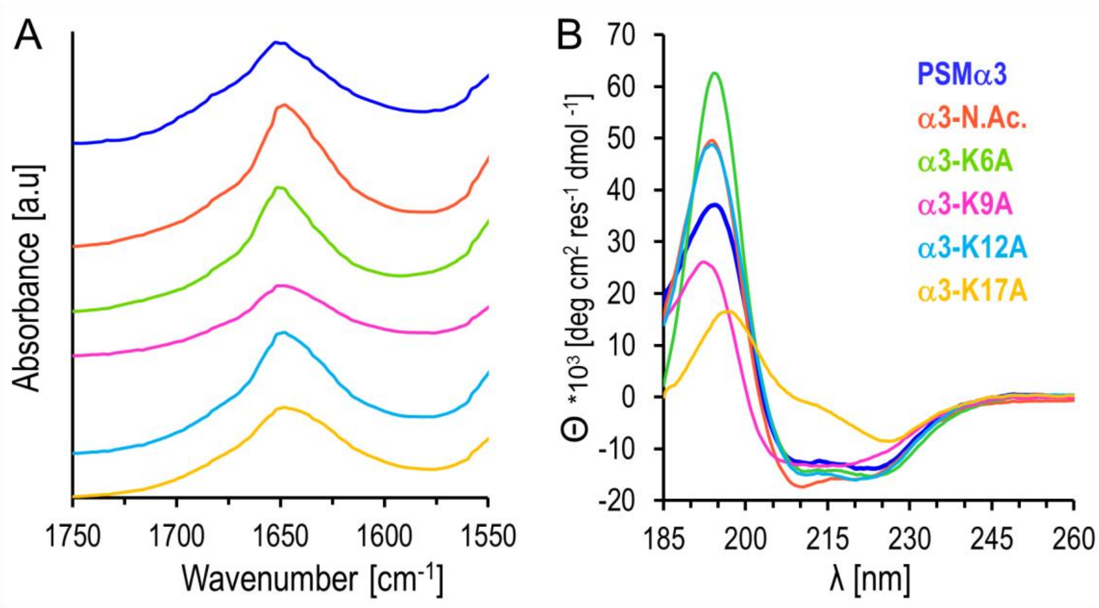

Figure S9. Circular Dichroism and FTIR spectroscopy of PSMα3 point mutated derivatives. A. Amide region FTIR spectra with typical α-helix maximum at 1650 cm^{-1}. **B.** CD spectra of the PSMα3 variants. The results are representative spectra of three independent repeats.

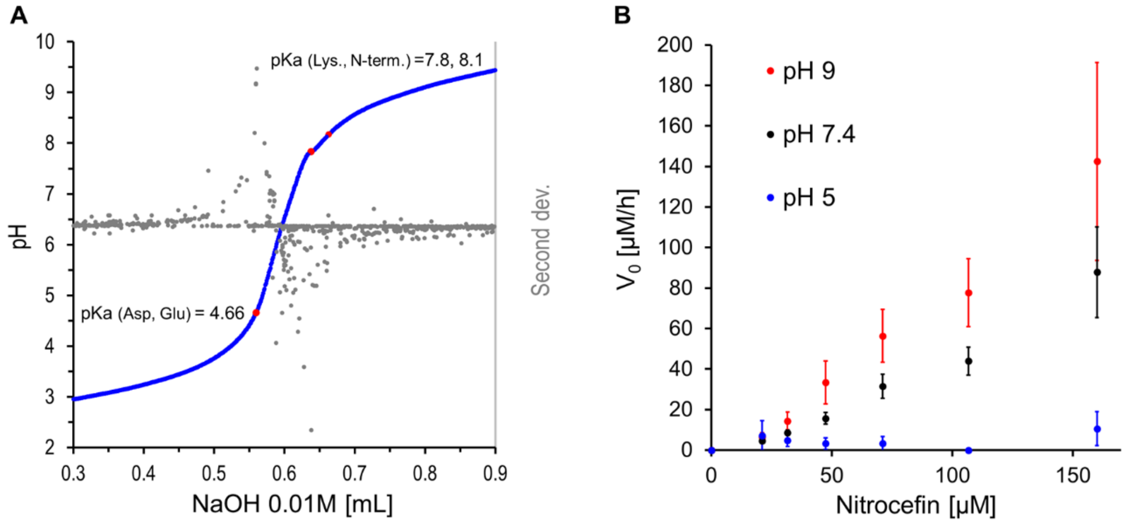

Figure S10. The effect of pH on PSMα3. A. Titration of PSMα3 preformed fibrils from pH=3 to pH=10. PSMα3 sample (400 μM) was preincubated as described above in DIW. After two hours of incubation the pH was dropped with HCl 1M and the sample was titrated in 1μL drops of NaOH 0.05M using automatic titrator, while measuring the pH. The result is representative result out of three independent repeats. **B.** Degradation of nitrocefin at pH 5,7.4 and 9 along 30 minutes in presence of PSMα3 preformed fibrils (50μM). PSMα3 fibril were prepared as described above. Then, the samples were diluted with the appropriate buffer (phosphate-citrate 50 mM for pH 5, HEPES 50 mM for pH 7.4 and 9), and nitrocefin was added at the appropriate concentration. The kinetics were measured as described earlier. Values are shown as average±SEM, N=3.

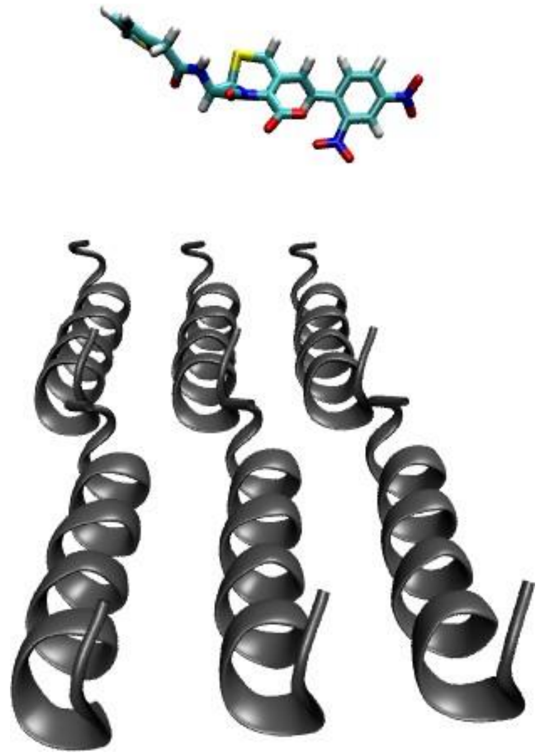

Figure S11. One of 4 Nitrocefin starting conformations for the Molecular Dynamics simulations. Nitrocefin was placed approximately 2 nm above six copies of PSMα3 arranged in a 3x2 fibril and extending infinitely in x-y through the periodic boundary conditions.

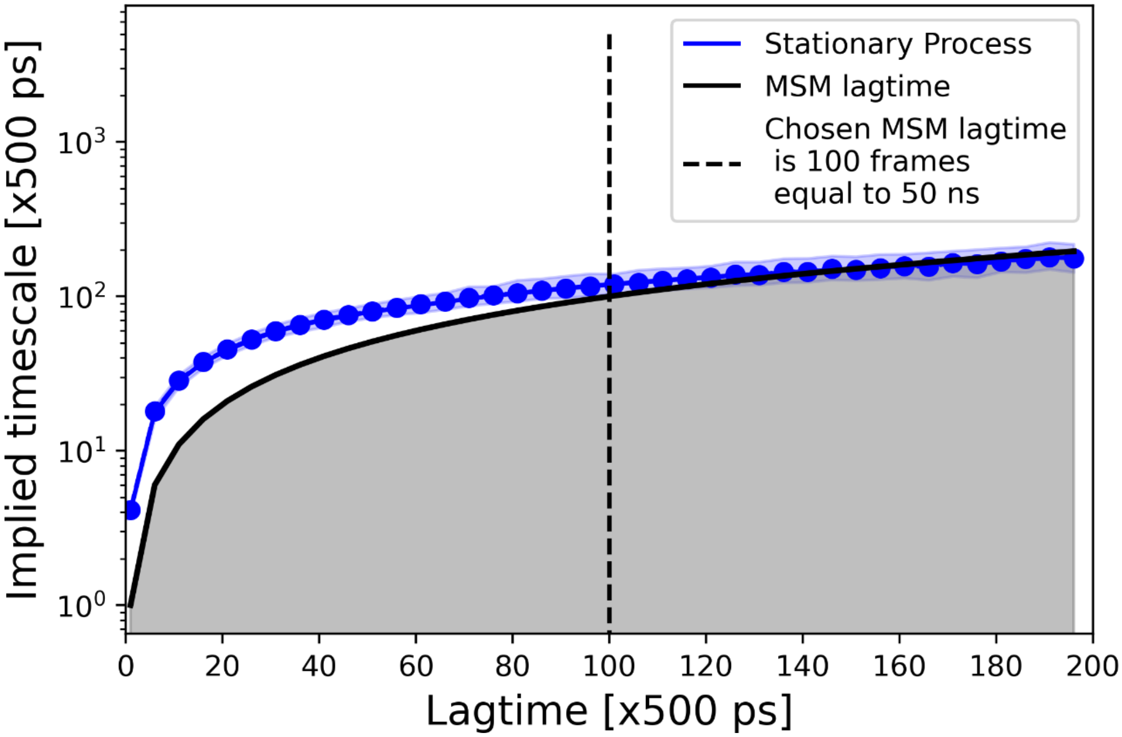

Figure S12. Implied timescale[3,4] of the stationary (equilibrium) process for a range of Bayesian Markov State Models with increasing lag times. The shaded area shows the error over 100 transition matrix samples. The implied timescale of the stationary process should be independent of the lag time in a Markovnian process. To find an appropriate lag time for our MSM, we thus choose the largest lag time where the implied timescale is approximately constant, but still above the lag time to resolve the process (blue line is above the black line).

Table S2. Gradient program for Amoxicillin

Step	Time, min	MP A, %	MP B, %
1	Initial	99	1
2	8	40	60
3	8.5	1	99
4	10	1	99
5	12	99	1
6	15	99	1

Table S3. Gradient program for Penicillin-G

Step	Time, min	MP A, %	MP B, %
1	Initial	90	10
2	5	40	60
3	7.5	40	60
4	10	10	90
5	12	10	90
6	13	90	10
7	15	90	10

The column temperature was kept at 40°C, the temperature of the samples was 10°C. The constant flow of 0.4mL/min was used. The total run time was 15 minutes. Mobile phase (MP) A was LCMS-grade water with 0.1% of LCMS-grade formic acid, MP B was LCMS-grade acetonitrile. The experiments were repeated in three independent experiments, and shown in one representative result.

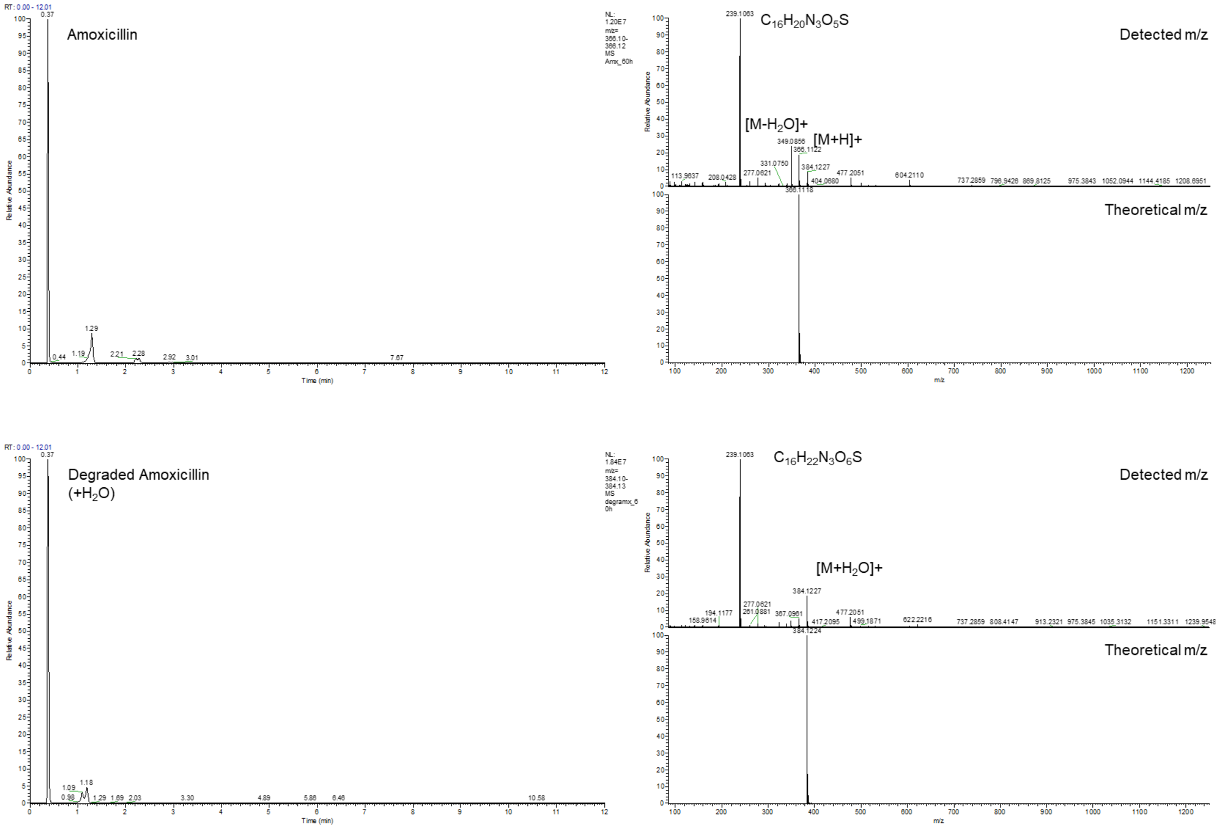

Figure S13. Amoxicillin and degraded Amoxicillin LC-MS chromatogram (left) and mass spectra (right)

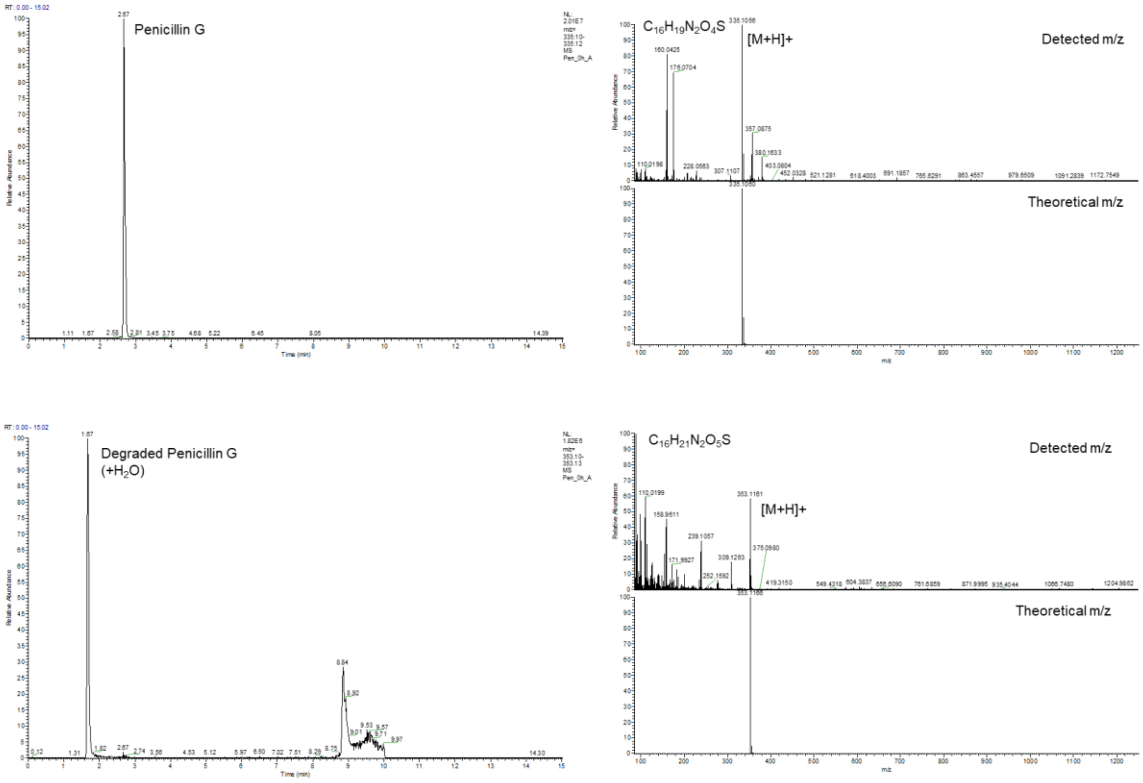

Figure S14. Penicillin-G and degraded Amoxicillin LC-MS chromatogram (left) and mass spectra (right)

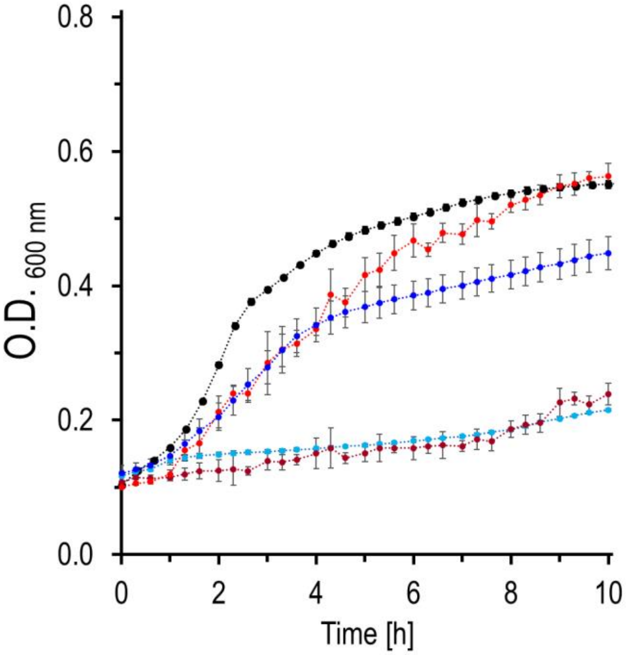

Figure S15. The effects of PSMα1 and PSMα4 upon nitrocefin in physiological scenarios. Growth curve of *S. aureus* (black), co-addition of preformed PSMα1 (blue), PSMα1 and 100 µg/mL nitrocefin (turquoise), PSMα4 (red) and co-addition of PSMα4 and 100 µg/mL nitrocefin (brown). PSMα1 and PSMα4 were preincubated in DIW and added to the growth media at a final concentration of 100 µM prior to addition of nitrocefin. Results are presented as an average ± SEM, N=4.

References:

1. Arad, E., Baruch Leshem, A., Rapaport, H. & Jelinek, R. β-Amyloid fibrils catalyze neurotransmitter degradation. *Chem Catal.* **1**, 908–922 (2021).

2. Schneider, A., Lang, A. & Naumann, W. Fluorescence spectroscopic determination of the critical aggregation concentration of the GnRH antagonists Cetrorelix, Teverelix and Ozarelix. *J. Fluoresc.* **20**, 1233–1240 (2010).

3. Trendelkamp-Schroer, B., Wu, H., Paul, F. & Noé, F. Estimation and uncertainty of reversible Markov models. *J. Chem. Phys.* **143**, 11B601_1 (2015).

4. Swope, W. C., Pitera, J. W. & Suits, F. Describing protein folding kinetics by molecular dynamics simulations. 1. Theory. *J. Phys. Chem. B* **108**, 6571–6581 (2004).
